# Alendronic acid modified PLGA drug delivery system loaded with 17β-Estradiol and vitamin D3 has anti-osteoporotic effect

**DOI:** 10.1016/j.mtbio.2026.102789

**Published:** 2026-01-12

**Authors:** Yonghui Wang, Sidi Zhang, Xinrun Ma, Donghao Hu, Junran Liu, Lu Wei, Xue Lei, Yan Hu, Fuyou Li, Yanhong Gao

**Affiliations:** a Department of Geriatrics, Xinhua Hospital, Shanghai Jiaotong University School of Medicine, Shanghai, China; b State Key Laboratory of Systems Medicine for Cancer, Shanghai Cancer Institute. Ren Ji Hospital School of Medicine, Shanghai Jiao Tong University, Shanghai, 200032, China; c Department of Chemistry, Fudan University, 2005 Songhu Road, Shanghai, 200438, China; d Department of Geriatrics, Shanghai General Hospital, Shanghai Jiaotong University School of Medicine, Shanghai, China; e School of Chemistry and Chemical Engineering & Institute of Translational Medicine, Shanghai Jiao Tong University, Shanghai, 200240, China

**Keywords:** Alendronic acid, 17β-Estradiol, Vitamin D3, Osteoporosis

## Abstract

Postmenopausal osteoporosis caused by estrogen deficiency often requires hormone replacement therapy (HRT), but its systemic side effects limit clinical application. Here, we developed a bone-targeted Poly (lactic-co-glycolic acid) (PLGA) nanocarrier modified with Alendronic acid (ADA) to co-deliver 17β-Estradiol (E2) and Vitamin D3 (VitD3), aiming to enhance efficacy and safety. The ADA-functionalized nanoparticles (E2+VD@PLGA_IR780_ADA) showed high drug loading (7.2 wt% for E2 and 2.3 wt% for VitD3), sustained release (>90 % over 48 h). In ovariectomized (OVX) mice, targeted delivery significantly improved bone mineral density, restored trabecular structure, and reduced serum bone resorption markers, while markedly alleviating E2-induced endometrial thickening. In vivo imaging confirmed selective bone accumulation. Mechanistically, co-administration of VitD3 and E2 elicits enhanced pro-osteogenic effects by virtue of VitD3-mediated Vitamin D Receptor (VDR) upregulation and amplified E2-induced estrogen receptor (ER) expression, which collectively drive robust activation of the PI3K/AKT/mTOR signaling cascade.This bone-specific nanoplatform offers a promising and safer strategy for osteoporosis therapy beyond conventional HRT.

## Graphical abstract

**Figure:**
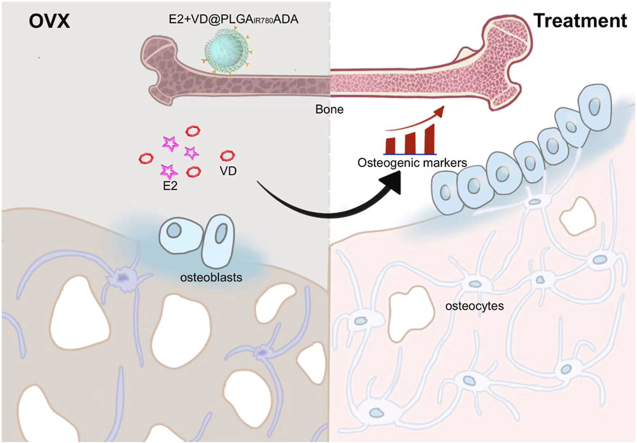
E2+VD@PLGA_IR780_ADA targets bones to improve osteogenesis.

## 1 Introduction

Postmenopausal osteoporosis, characterized by accelerated bone resorption and microarchitectural deterioration, affects over 200 million women globally and imposes a heavy socioeconomic burden due to fracture-related morbidity [[1], [2], [3], [4], [5], [6]]. Estrogen deficiency—the primary driver of postmenopausal bone loss—disrupts osteoblast-osteoclast equilibrium, leading to uncoupled bone remodeling [[7], [8], [9], [10], [11]]. While hormone replacement therapy (HRT) with 17β-Estradiol (E2) effectively mitigates bone loss and alleviates menopausal symptoms [[12], [13], [14], [15], [16]], its systemic administration raises concerns over endometrial hyperplasia, thromboembolism, and breast cancer risks [], severely limiting long-term clinical utility.

Vitamin D3 (VitD3), a key regulator of calcium homeostasis, facilitates E2 to enhance osteoblast differentiation and calcium absorption [[17], [18], [19], [20]]. However, the hydrophobicity of both E2 and VitD3 necessitates high-dose administration, exacerbating off-target effects [21]. Poly (lactic-co-glycolic acid) (PLGA)-based nanoparticles (PLGANPs) have emerged as promising carriers for hydrophobic drugs due to their biodegradability and controlled release profiles [22]. Despite these advantages, conventional PLGA systems lack tissue specificity, resulting in suboptimal drug accumulation at bone sites and limited therapeutic efficacy [[[23], [24]]. Recent advances in bone-targeted drug delivery highlight the potential of bisphosphonate ligands (e.g., alendronic acid, ADA) to enhance hydroxyapatite-binding affinity. For instance, Chen et al. demonstrated that ADA-modified liposomes achieved 3.8-fold higher bone accumulation than non-targeted counterparts [25], while Zheng et al. reported ADA-functionalized exosomes for osteoporosis therapy [26]. Nevertheless, simultaneous co-delivery of E2 and VitD3 via bone-targeted PLGA NPs remains unexplored—a critical gap given their osteogenic effects.

To address these challenges, we designed a multifunctional PLGA-based nanoplatform (E2+VD@PLGA_IR780_ADA) that integrates three innovative features: (1) bone-targeted delivery via ADA modification to enhance hydroxyapatite affinity and minimize off-target exposure, (2) co-encapsulation of E2 and VitD3 within a single carrier to amplify osteogenic differentiation, and (3) real-time biodistribution tracking enabled by IR780 fluorescence for non-invasive pharmacokinetic monitoring. Unlike previous PLGA systems limited by passive diffusion or single-drug loading [[27], [28]], our design leverages ADA-mediated active targeting to achieve localized drug accumulation in bone tissue, while the dual-drug payload capitalizes on the complementary roles of E2 (inhibiting osteoclastogenesis) and VitD3 (promoting osteoblast mineralization). Furthermore, the incorporation of IR780 not only facilitates in vivo imaging but also ensures batch-to-batch consistency through quantifiable fluorescence signals—a feature absent in conventional “stealth” nanocarriers.

This study demonstrates that E2+VD@PLGA_IR780_ADA significantly improves bone mineral density (BMD) in ovariectomized (OVX) mice while attenuating uterine hyperplasia, offering a paradigm-shifting strategy for precision osteoporosis management. By unifying targeted delivery, pharmacology, and traceable pharmacokinetics, our platform overcomes the limitations of traditional HRT and paves the way for safer, more effective nanomedicine-based interventions in bone disorders.

## 2 Materials and methods

### 2.1 Preparation of the IR780-labeled PLGA polymer nanocarrier (PLGA_IR780_)

The PLGA polymer (2.0 mg) was dissolved in 1.0 mL of Dimethyl Formamide (DMF). After that, 2 mL aqueous solution of IR780 (0.50 mg/mL) was added dropwise into the solution under stannous stirring to form IR780-labeled PLGA (PLGA_IR780_). The residual DMF was removed by dialysis against deionized water for two days. The entire process was conducted in the absence of light.

### 2.2 Preparation of drug-loaded polymer nanocapsules

E2 (1.51 mg) and VitD3 (0.5 mg) were dissolved in 1 mL nujol to obtain stock A. Then, stock A solution (40 μL) was added to PLGA_IR780_ (5 mg) dissolved in dichloromethane (200 μL), into which 5 % polyvinyl alcohol (PVA) solution (w/v) (1.26 mL) was added. After being well blended, the mixture was sonicated using a sonicator equipped with a microtip in an ice water bath for 5 min in total. Then, another 0.6 mL of PVA solution was added to homogenize the emulsion, which was subsequently stirred for 4 h at room temperature to evaporate the organic solvents. The product was then centrifuged at 14,800 rpm for 10 min at 4 °C and washed several times with purified water to remove the PVA. The final product of the drug-loaded polymer nanocapsule (E2+VD@PLGA_IR780_) was collected and stored in deionized water at 4 °C for future use.

### 2.3 Synthesis of ADA-PLGA

First, 1 mg/mL E2+VD@PLGA_IR780_, 0.8 mg of N-hydroxysuccinimide (NHS) and 1.9 mg of ethyldimethylaminopropyl carbodiimide (EDC) were dissolved in 5 mL of dichloromethane and stirred for 24 h at room temperature to convert carboxylated PLGA (PLGA-COOH) to amide-activated PLGA (PLGA-NHS). Then, 10 mg of ADA was added to the solution and stirred for 8 h. Dichloromethane was subsequently removed using a rotary evaporator, and 5 mL of DMF was added to dissolve the product. After that, the solution was added to a dialysis bag (width of 44 mm, and the molecular weight cutoff was 8000–14,000), and unreacted ADA and other small molecules were removed by dialyzing for 3–5 days. The product was subsequently freeze-dried for 48 h to obtain E2+VD@PLGA_IR780_ADA.

### 2.4 E2 and VitD3 release capability in PLGA_IR780_ADA

The release capability of E2 or VitD3 from drug-loaded polymer nanocapsules were characterized. The polymer nanocapsules (2 mg/mL) were dispersed in 3 mL of phosphate-buffered saline (PBS, pH 7.4) at 37 °C. A small aliquot of the solution was taken at given time intervals, and the concentrations of the supernatants containing released E2 or VitD3 were determined by ultraviolet visible (UV–vis) spectroscopy.

### 2.5 Cell culture

MC3T3-E1 cells were purchased from Shanghai Institutes for Biological Sciences (Shanghai, China) and cultured in Dulbecco's modified Eagle's medium (DMEM, Gibco, Thermo Fisher Scientific, USA) supplemented with 10 % fetal bovine serum (FBS, Gibco, USA), 100 U/ml penicillin and 100 mg/mL streptomycin (Gibco, USA). The cells were cultured in an incubator with 5 % CO_2_ at 37 °C.

### 2.6 In vitro cytotoxicity test

Cell viability was quantitatively evaluated by methyl thiazolyl tetrazolium (MTT, Sigma-Aldrich, USA) assays. Briefly, MC3T3-E1 cells were seeded into a 96-well cell culture plate at 1 × 10^4^ cells per well and cultured at 37 °C and 5 % CO_2_ for 24 h. After that, the cells were treated with different concentrations of E2+VD@PLGAIR780ADA and further cultured for another 24 h. MTT (10 μL, 5 mg/mL) was subsequently added to each well, and the cells were incubated for an additional 4 h. Desorption medium and DMSO were added to each well (100 μL total volume). The optical density (OD490) value (Abs.) of each well was detected with a multimode microplate reader (BioTek). The cell viability (%) was calculated according to previous reports:

$$\text{Cell}\;\text{viability}\;\left(\%\right)=\left(\frac{\mathrm{Mean}\;\mathrm{of}\;\mathrm{Abs}:\mathrm{value}\;\mathrm{of}\;\mathrm{treatment}\;\mathrm{group}}{\mathrm{Mean}\;\mathrm{Abs}:\mathrm{value}\;\mathrm{of}\;\mathrm{control}}\right)\times 100\%$$

### 2.7 Uptake test in MC3T3-E1 cells

Approximately 1 × 10^5^ MC3T3-E1 cells were seeded in each well of a 6-well cell culture plate and incubated overnight. Then, E2+VD@PLGA_IR780_ADA or PBS was added, and the MC3T3-E1 cells were incubated for another 4 h. Then, the cell culture medium was removed, and the cells were washed with PBS several times. The slides were mounted and then observed under a confocal microscope (Olympus FV1000, designed by our group).

### 2.8 In situ and ex vivo imaging

The animal procedures were performed in accordance with the guidelines of the Institutional Animal Care and Use Committee. In situ and ex vivo imaging studies were carried out with a modified Kodak in vivo imaging system consisting of an external 0–5 W adjustable CW infrared laser (730 nm, Connect Fiber Optics, China) as the excitation source and an Andor DU897 EMCCD as the signal collector. The emission signals were measured at 780 nm. Images of the signals were analyzed with Carestream MI SE. BALB/c mice (4 weeks old) were injected intraperitoneally with E2+VD@PLGA_IR780_ADA (400 μL, 2.5 mg/mL) in PBS. The control group was injected with PBS (400 μL). At 6 h postinjection, we carried out whole-body imaging without the major organs to expose the bones. After that, bones (arm, leg, sternum, and spine) and the main organs (liver, kidney, spleen, heart, lungs, fallopian tube) were removed for ex vivo imaging. All mentioned animal work was approved by the Ethics Committee of Xinhua Hospital Affiliated with Shanghai Jiao Tong University School of Medicine.

### 2.9 ALP staining and ALP activity

To induce osteogenesis in MC3T3-E1 cells, when the cells reached 70–80 % confluence, complete medium supplemented with 50 μg/mL ascorbic acid (Sigma-Aldrich, USA), 100 nM dexamethasone (Sigma-Aldrich, USA) and 10 mM β-glycerophosphate (Sigma-Aldrich, USA) was added. The AKT inhibitor (Akti1/2, MedChemExpress, USA) was dissolved in DMSO and used at a final concentration of 10 μM. The medium was changed every 3 days. Alkaline Phosphatas (ALP) staining was performed on the 7th day of induction, after which the cells were fixed with 4 % Paraformaldehyde (PFA, Beyotime, China), washed with PBS, and stained with the mixture according to the instructions (Beyotime, China). ALP activity was measured on the 7th day of osteogenic induction, the cells were lysed with RIPA buffer, the protein concentration was evaluated with a BCA protein assay (Beyotime, China), and the ALP concentration was measured with an alkaline phosphatase assay kit (Beyotime, China). ALP activity was the amount of ALP normalized to the total protein amount.

### 2.10 Alizarin Red (ARS) staining

After 21 days of induction with osteogenic medium, the cells were fixed with 4 % PFA for 15 min at room temperature. Then, the cells were washed with PBS 3 times and stained with 2 % Alizarin red S solution (Sigma-Aldrich, USA) for 30 min at room temperature. The cells were washed 3 times after staining. Mineral nodules were observed by light microscopy (Leica DMI 3000B, Germany).

### 2.11 qRT-PCR

Total RNA was extracted from cells with Trizol (Takara Biotechnology, Japan). To make the templates for qRT-PCR, cDNA was generated from total RNA by reverse transcription with a PrimeScript™ RT Master Mix (Takara Biotechnology, Japan). Then, qRT-PCR analysis was performed with 2 × SYBR master mix (Yeason, China). The relative standard curve method (2^-△△CT^) was used to determine the relative mRNA expression, which was normalized to the housekeeping gene GAPDH. Primer sequences for qRT-PCR analysis are provided in Supplementary Table.

### 2.12 Immunohistochemical (IHC) staining

MC3T3-E1 cells were induced to undergo osteogenesis for 14 days. Then, the cells were fixed with 4 % PFA for 20 min. Nonspecific antigens were blocked and permeabilized with 10 % Bovine Serum Albumin (BSA, Beyotime, China) containing 0.5 % Triton X-100 (Beyotime, China) for 1 h at room temperature. The cells were incubated with primary antibodies against osteopontin (OPN, Rabbit, 1:200, Proteintech, China) and osteocalcin (OCN, Rabbit, 1:200, Proteintech, China) overnight at 4 °C, followed by incubation with a goat anti-rabbit streptavidin‒horseradish peroxidase (HRP)-conjugated secondary antibody (1:1000, Proteintech, China) for 1 h at room temperature. The presence of the expected protein was visualized by diaminobenzidine (DAB) staining (Beyotime, China), and the nuclei were visualized under a microscope (Leica DMI 3000B, Germany).

### 2.13 Effect of E2+VD@PLGA_IR780_ADA in OVX mice

Thirty-two 8-week-old female Kunming mice were purchased from Shanghai Laboratory Animal Centre (Shanghai, China). The mice were housed in an air-conditioned room at 23–25 °C with a 12 h light/dark cycle and had free access to water and were fed a standard diet. All the animal experiments were approved by the Ethics Committee of Xinhua Hospital Affiliated with the Shanghai JiaoTong University School of Medicine. All the experimental procedures were carried out in accordance with the Regulations for the Administration of Affairs Concerning Experimental Animals. Young mature female Kunming mice that underwent bilateral ovariectomy (OVX) or sham operations (Sham) at 6 weeks of age were used. The mice were assigned to 4 groups (*n* = 8 per group): the Sham + PBS, OVX + PBS, OVX + E2+VD, and OVX + E2+VD@PLGA_IR780_ADA groups. They were allowed to acclimate in the Animal Research Facility for 2 weeks after surgery. The treatments were given as intraperitoneal injections every 2 days for 8 weeks. The concentration of E2 was 50 μg/kg, and the concentration of VitD3 was 1.5 μg/kg. After 8 weeks of treatment, the mice were sacrificed, and the legs, spine and blood were collected.

### 2.14 Micro-CT

To test the microstructure of the bone, the fourth lumbar (L4) vertebra of each mouse was measured using a Bruker Micro-CT SkyScan 1276 system (Kontich, Belgium). The scan settings were as follows: voxel size, 6.534165 μm; medium resolution, 70 kV; 200 μA; 1 mm Al filter; and integration time, 350 ms. The density measurements were calibrated to the manufacturer's calcium hydroxyapatite (CaHA) phantom. The 3D images and the bone morphometric parameters of bone volume over total volume (BV/TV), trabecula number (Tb.N), trabecula separation (Tb.Sp) and bone mineral density (BMD) were determined by analyzing the regions of interest (ROIs) using a CT Analyzer (1.20.3.0).

### 2.15 Three-point bending test

To measure the bone strength of the tibia, a small animal bone strength test instrument (YLS-16A, Corp., Yanyi Jinan, P. R. China) was used to perform the three-point bending test. Fresh humeri were separated from the mice, and the tests were performed immediately. Two end support points and one central loading point were used in the three-point bending test. The biomechanical measurement data were collected from the load‒deformation curves. The maximum load (N) was recorded.

### 2.16 H&E staining

The tissues were fixed in 4 % PFA at 4 °C for 48 h, and the femurs were decalcified in 10 % EDTA for 4 weeks. Fixed femurs were embedded in paraffin for histological analysis. For histological studies, Hematoxylin&Eosin (H&E) staining was used. All histological determinations were carried out on sagittal 5–7 mm thick sections/sample.

### 2.17 ELISA

Sera were collected from the mice and frozen at −80 °C. Serum C-terminal telopeptide of collagen I (CTX-I) and serum osteocalcin (OCN) were measured using enzyme-linked immunosorbent assay (ELISA) kits (Westang Biotech, China) following the manufacturer's instructions.

### 2.18 Western blot

Cells were lysed in cold RIPA buffer supplemented with protease and phosphatase inhibitors. The concentration of the sample was measured with a BCA protein assay kit (Beyotime, China) following the manufacturer's instructions. The samples were loaded onto SDS-polyacrylamide gels, and proteins were transferred to PVDF membranes. The membranes were incubated with primary antibody (ERα, ERβ, VDR, P-PI3K, Proteintech, 1:1000; PI3K, Abcam, 1:1000; P-Akt, Akt, mTOR, P-mTOR, FOXO3, P-FOXO3, Proteintech, 1:1000) at 4 °C overnight, followed by incubation with an HRP-conjugated secondary antibody (HRP-labeled Goat Anti-Rabbit IgG, Proteintech, 1:2000; HRP-labeled Goat Anti-Mouse IgG, Proteintech, 1:2000) at 37 °C for 1 h. The immunoreactive bands were visualized using an enhanced chemiluminescence reagent (Thermo Fisher Scientific, USA) and imaged with a ChemiDocTM MP Imaging System (Bio-Rad, USA).

### 2.19 Statistics

The experimental results are expressed as the means ± standard deviations (SDs). Student's two‐tailed t‐test and one‐way ANOVA were performed using GraphPad Prism 8 software (GraphPad, San Diego, CA, USA). A p ≤ 0.05 was considered statistically significant.

## 3 Results and discussion

### 3.1 Synthesis and characterization of E2+VD@PLGA_IR780_ADA

Bone-targeting polymer nanocapsules were prepared in three steps, as shown in Fig. 1. First, PLGA and IR780 were mixed in DMF, and the DMF was removed with water through a dialysis process to obtain the IR780-labeled PLGA (PLGA_IR780_). As shown in Fig. 2A, intense luminescence of PLGA_IR780_ was successfully acquired using a fluorescence spectrophotometer. Two types of nanosystems (nanosystems with and without ADA, hereafter referred to as E2+VD@PLGA_IR780_ and E2+VD@PLGA_IR780_ADA, respectively) were subsequently synthesized and assessed for their ability to load E2 and VitD3 and target bone tissue. PLGA_IR780_ containing E2 and VitD3 nanosystems was prepared using a double emulsion (water/oil/water) solvent evaporation process, and ADA units were modified on the surface of the nanoparticles by an amidation procedure. E2+VD@PLGA_IR780_ and E2+VD@PLGA_IR780_ADA were characterized via a combination of transmission electron microscopy (TEM), dynamic light scattering (DLS), UV–vis spectroscopy and Fourier Transform Infrared (FTIR) spectroscopy.

**Fig. 1:**
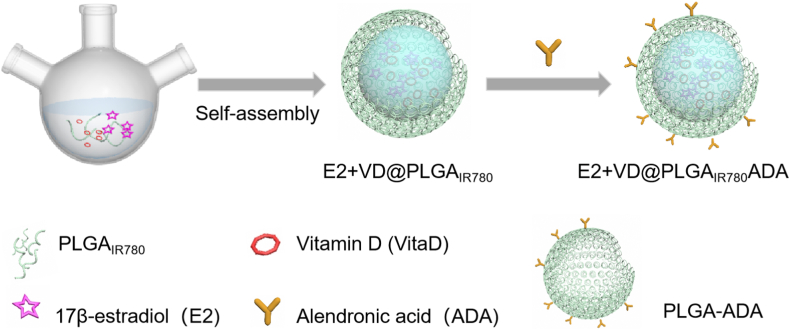
Schematic illustration of the synthesis of the E2+VD@PLGA_IR780_ADA nanosystem.

**Fig. 2:**
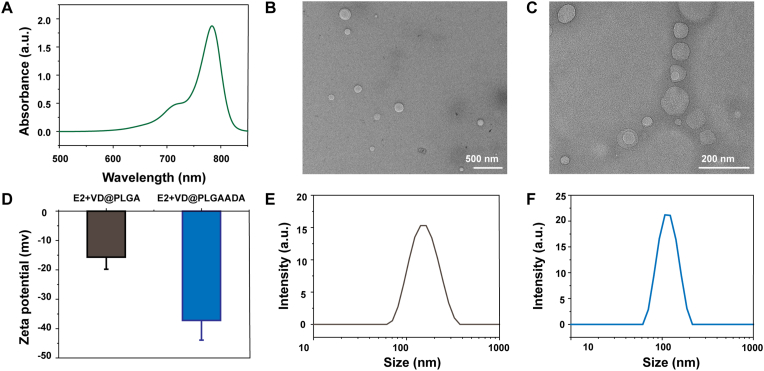
(A) UV–visible absorbance spectra of PLGA_IR780_. (B, C) Typical morphologies of E2+VD@PLGA_IR780_ and E2+VD@PLGA_IR780_ADA confirmed by TEM. (D) Zeta potentials of E2+VD@PLGA_IR780_ and E2+VD@PLGA_IR780_ADA. (E, F) Size distributions of E2+VD@PLGA_IR780_ and E2+VD@PLGA_IR780_ADA as measured via DLS.

The morphologies of E2+VD@PLGA_IR780_ and E2+VD@PLGA_IR780_ADA exhibited regular spherical shapes, as confirmed by TEM (Fig. 2B and C), and the surface charges of E2+VD@PLGA_IR780_ and E2+VD@PLGA_IR780_ADA were −15.7 ± 4.1 mV and −37.2 ± 6.7 mV, respectively (Fig. 2D). Additionally, the hydrodynamic diameters of E2+VD@PLGA_IR780_ and E2+VD@PLGA_IR780_ADA were 242 ± 6.5 nm and 175 ± 10.4 nm, respectively, as measured by DLS, which was consistent with the TEM results (Fig. 2E and F). To further confirm the existence of ADA, the chemical structures of the nanosystems without the IR780 label (named E2+VD@PLGA and E2+VD@PLGAADA) were confirmed by FTIR spectroscopy, and two groups of bands in the range of 1800–1200 cm^−1^ (P–O stretching) and 670–500 cm^−1^ (O–P–O bending) due to [PO_3_]^2-^ vibrations corresponding to ADA were clearly observed, confirming the presence of ADA groups in E2+VD@PLGA_IR780_ADA (Fig. 3).

**Fig. 3:**
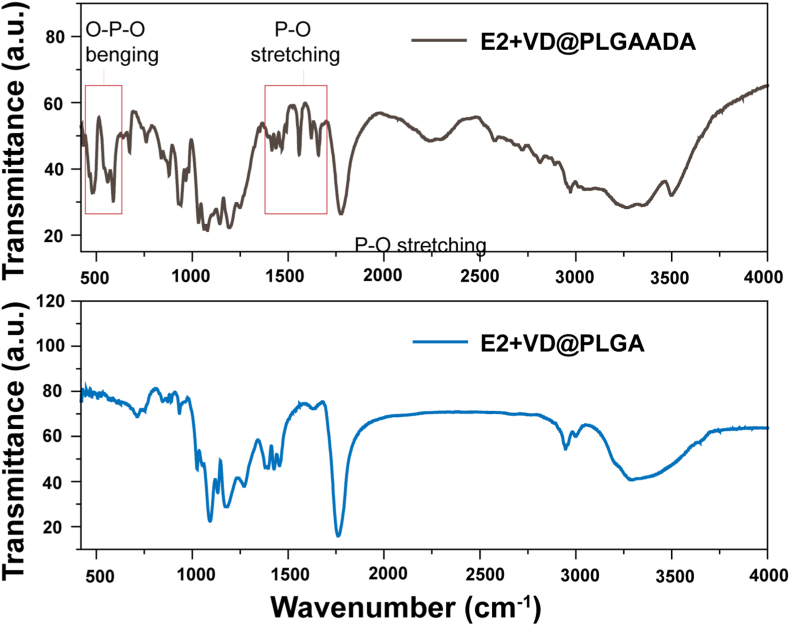
FTIR spectra of E2+VD@PLGAADA and E2+VD@PLGA.

The loading efficiencies of E2 and VitD3 were subsequently determined by UV–vis spectroscopy. As shown in Fig. 4A and C and Fig. S1, the loading efficiency of E2 was calculated to be 7.2 wt%. The VitD3 content was calculated to be 2.3 wt%, excluding E2 (Fig. 4B and C and Fig. S2). The release behaviors of E2 and VitD3 from E2+VD@PLGA_IR780_ADA were investigated in a PBS solution at pH 7.4 (Fig. 4D). A rapid release rate was observed at 12 h, resulting in the delivery of more than 50 % of the loaded drug. The subsequent release rate was slow, and >90 % of the drug was released after 48 h. The size of E2+VD@PLGA_IR780_ADA was reduced from 230 ± 10.6 to 56 ± 5.9 nm, and the TEM image revealed further expansion of the nanoparticles into polymersomes (Fig. S3). These results collectively suggest that these nanocapsules can be effectively used as sustained release carriers for therapeutic hormones.

**Fig. 4:**
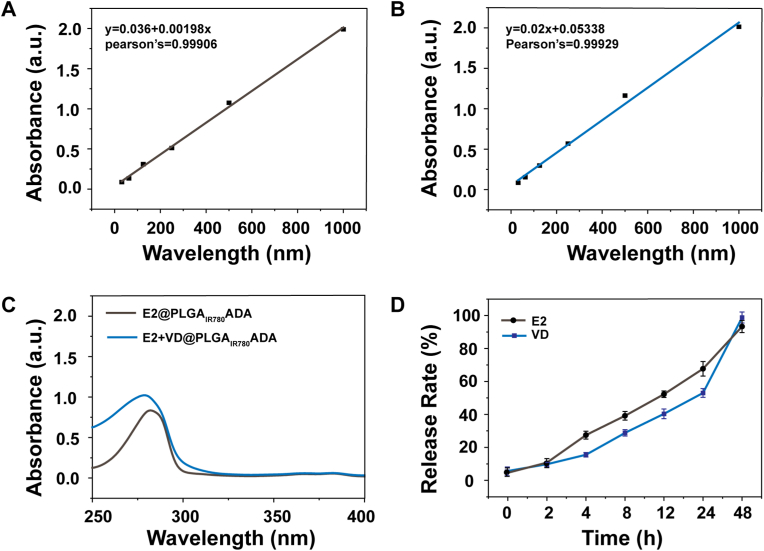
(A, B) Absorbances at 278 nm and 265 nm as a function of the E2 and VD concentrations. (C) UV–visible absorbance spectra of E2@PLGA_IR780_ADA and E2+VD@PLGA_IR780_ADA. (D) Release behaviors of E2 and VD from E2+VD@PLGA_IR780_ADA in PBS.

### 3.2 Tracking of E2+VD@PLGA_IR780_ADA in vitro

ADA is an FDA-approved bisphosphonate that prevents bone resorption by inhibiting osteoclast activity and increasing osteoclast death [29]. In addition, ADA has a high affinity for the bone surface; thus, it is widely applied in drug delivery systems. In 2018, Chen and colleagues reported that ADA modification significantly increased the mineral binding affinity of liposomes and facilitated gene delivery to osteoblastic cells [30]. Another study demonstrated that the in vitro hydroxyapatite binding affinity and in vivo bone-targeting aggregation of exosomes were significantly improved after ADA modification [26]. An effective nanocarrier should release E2 and VitD3 precisely to minimize their side effects and protect their activity before arriving at the action sites. The MTT assay provides a sensitive means to evaluate osteoblast proliferation because cell viability is measured based on a determination of the dehydrogenase activity in mitochondria. As shown in Fig. S4, treatment with E2, the nanocapsule, or E2+VD@PLGA_IR780_ADA did not reduce the viability of MC3T3-E1 cells after 48 h of treatment. We subsequently evaluated the cellular uptake of E2+VD@PLGA_IR780_ADA using confocal microscopy. As shown in Fig. S5, bright fluorescent dots were observed in the cytoplasm of MC3T3-E1 cells incubated with E2+VD@PLGA_IR780_ADA, indicating that E2+VD@PLGA_IR780_ADA has a unique advantage in drug release tracking.

### 3.3 E2+VD@PLGA_IR780_ADA improves osteogenesis in MC3T3-E1 cells

To determine the osteogenic effect of E2+VD@PLGA_IR780_ADA in MC3T3-E1 cells, we first performed ALP staining and evaluated the corresponding ALP activity. As shown in Fig. 5A and B, the ALP staining and activity of E2+VD were greater than those of the individual components, E2 (10^−7^ mol/L) and VitD3 (10^−6^ mol/L), after 7 days of induction. ALP staining and activity in the E2+VD@PLGA_IR780_ADA group were stronger than those in the E2+VD group, whereas the ALP activity levels in the control and PLGA-ADA groups rarely changed. We subsequently measured the mRNA expression levels of the osteogenic markers Runx2, ALP and IBSP, which are indicators of early osteogenic differentiation ability (Fig. 5C). The qRT-PCR results revealed that either E2 or VitD3 treatment increased the mRNA expression levels of osteogenic markers. However, the combination of E2 and VitD3 improved osteogenesis, resulting in increased expression levels of osteogenic markers. Similarly, E2+VD@PLGA_IR780_ADA induced greater osteogenic differentiation in MC3T3-E1 cells than in the control, PLGA-ADA, E2 and VD groups after 7 days of osteogenesis. Next, we examined the ability of the nanoparticles to induce late osteogenic differentiation by ARS staining and IHC. The protein expression levels of OPN and OCN, which are late-stage osteogenesis markers, in MC3T3-E1 cells were measured by IHC staining after 14 days of osteogenic induction (Fig. 5D). ARS staining was used to assess calcium deposition, which is a characteristic of mature osteoblasts after 21 days of differentiation (Fig. 5E). There were more red-stained mineral nodules in the E2+VD@PLGA_IR780_ADA and E2+VD groups than in the other groups. Similar to the results of IHC staining, the E2+VD@PLGA_IR780_ADA and E2+VD groups presented the strongest activity levels compared with the other groups.

**Fig. 5:**
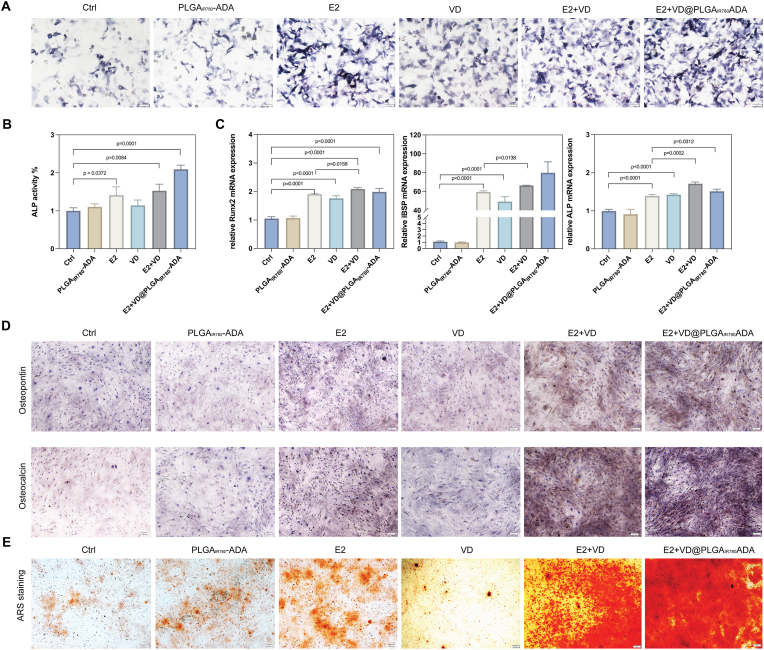
E2+VD@PLGA_IR780_ADA improved osteogenesis in vitro. (A) ALP staining images of MC3T3-E1 cells induced with PBS, PLGA-ADA, E2, VD, E2+VD or E2+VD@PLGA_IR780_ADA for 7 days. (B) ALP activity was quantified after 7 days of induction. (C) Q‒PCR analysis of Runx2, IBSP, and ALP in MC3T3-E1 cells after 7 days of osteogenic induction with PBS, PLGA-ADA, E2, VD, E2+VD or E2+VD@PLGA_IR780_ADA. (D) Immunohistological staining of osteopontin and osteocalcin in MC3T3-E1 cells induced with PBS, PLGA-ADA, E2, VD, E2+VD or E2+VD@PLGA_IR780_ADA for 14 days. (E) Alizarin Red S-stained images of MC3T3-E1 cells induced with PBS, PLGA-ADA, E2, VD, E2+VD or E2+VD@PLGA_IR780_ADA for 21 days. (n = 3). (For interpretation of the references to colour in this figure legend, the reader is referred to the Web version of this article.)

### 3.4 E2+VD@PLGA_IR780_ADA improves bone mass and strength in OVX mice

To reveal the therapeutic effect of E2+VD@PLGA_IR780_ADA in OVX mice, which constitute a postmenopausal osteoporosis model, the mice were intraperitoneally injected with PBS, E2+VD, or E2+VD@PLGA_IR780_ADA (with an E2 concentration of 50 μg/kg and a VitD3 concentration of 1.5 μg/kg) every 2 days. The lower limbs were subsequently collected for microCT, H&E, and ELISA to examine the bone volume, bone histology, and serum turnover markers in the different groups. Images of the micro-CT images of the Sham + PBS, OVX + PBS, OVX + E2+VD, and OVX + E2+VD@PLGA_IR780_ADA groups are shown in Fig. 6A; less trabecular bone was observed in the OVX + PBS groups than in the other groups, which indicates that OVX procedure successfully caused bone loss, and more trabecular bone was generated in the OVX + E2+VD@PLGA_IR780_ADA group, which indicates the potential rescuing effect of OVX + E2+VD@PLGA_IR780_ADA. Surprisingly, OVX mice treated with the combination of E2 and VitD3 presented more trabecular bone than OVX + PBS mice did, confirming the better osteogenic effect of these two components. The microstructure indices were analyzed to provide more detail on the treatment effects (Fig. 6B). In the OVX + E2+VD@PLGA_IR780_ADA group, the BMD increased by approximately 50 %, the BV/TV increased by approximately 50 %, and the Th.N was increased by 25 %, and the Th.Sp was decreased by approximately 20 % compared with that in the OVX + PBS group, as shown in Fig. 6B. Similarly, the BMD and BV/TV were greater and the Th.Sp was lower in the OVX + E2+VD group than in the OVX + PBS group. However, the microstructure indices did not differ between the OVX + E2+VD and OVX + E2+VD@PLGA_IR780_ADA groups. These results were consistent with the H&E staining of the proximal ends of the tibias (Fig. 6C). For analysis of bone stability, the tibias were subjected to 3-point bending tests. As shown in Fig. 6D, the maximum bending force was distinctly lower in the OVX + PBS group than in the Sham + PBS group, which indicated that OVX led to greater bone fragility. When E2+VD or E2+VD@PLGA_IR780_ADA was applied to OVX mice, the maximum bending force improved compared with that in OVX + PBS mice and recovered to the extent of that in Sham + PBS mice. However, the bending force following E2+VD@PLGA_IR780_ADA treatment was not significantly greater than that of OVX + PBS mice. The cells residing inside bones secrete bone turnover factors, which are indicators of bone remodeling. An analysis of the concentrations of CTX-1 and OCN in mouse serum by ELISA, as shown in Fig. 6E, revealed that the concentrations of CTX-1 and OCN in the OVX + PBS group were much greater than those in the Sham + PBS group, and these concentrations are released by resorbed bone, which is consistent with active bone resorption activity in osteoporosis. Interestingly, the concentrations of CTX-1 and OCN in the E2+VD group were much lower than those in the OVX + PBS group. However, the decrease in the concentration of CTX-1 in the OVX + E2+VD@PLGA_IR780_ADA group was not statistically significant, which might be the result of high deviation. The concentration of OCN decreased significantly in the OVX + E2+VD@PLGA_IR780_ADA group. The serum concentrations of CTX-1 and OCN were comparable in the OVX + E2+VD and OVX + E2+VD@PLGA_IR780_ADA groups. These results indicate that E2+VD@PLGA_IR780_ADA treatment can reverse the bone loss caused by osteoporosis.

**Fig. 6:**
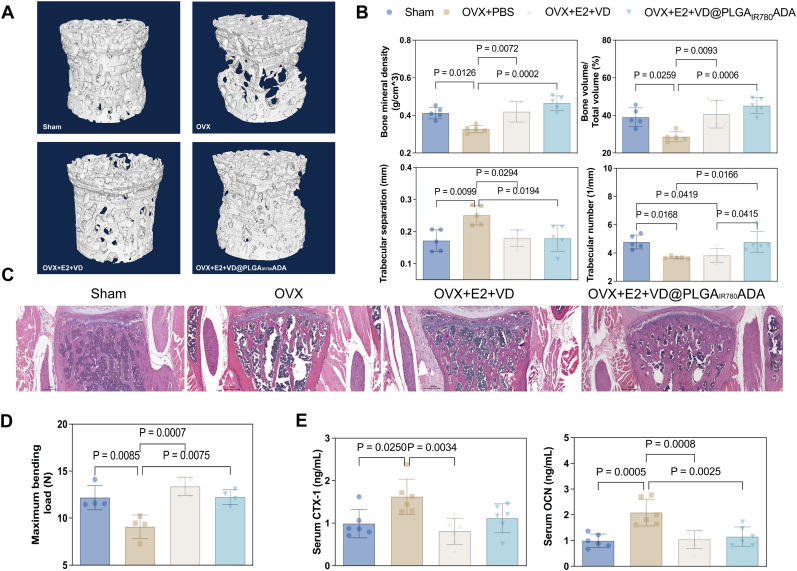
Effects of E2+VD@PLGA_IR780_ADA in OVX mice. (A) Three-dimensional reconstructed images of trabecular bone from the fourth lumbar vertebra of the Sham + PBS, OVX + PBS, OVX + E2+VD, and OVX + E2+VD@PLGA_IR780_ADA groups. (n = 5) (B) H&E staining of the lumbar vertebrae. (C) Quantitative analysis of BMD, BV/TV, Tb.Sp, and Tb.N. (n = 5) (D) Results of the biomechanical test of the maximum bending load. (n = 4) Serum levels of the bone turnover markers OCN and CTX-1 in the four groups. (n = 6) (E) Serum levels of the bone turnover markers OCN and CTX-1 in the four groups. (n = 6).

VitD3 and E2 were well acknowledged osteogenic inducers applied in clinical treatment of osteoporosis. In our study, both VitD3 and E2 were capable of improving the osteogenesis of MC3T3-E1 cells, with increased mRNA expression levels of Runx2, ALP and IBSP and increased protein levels of OPN and OCN detected by ARS staining. When VitD3 was combined with E2 without a delivery system, ALP activity, the mRNA expression levels of early osteogenic markers, calcium deposits, and the protein levels of late osteogenic markers were markedly greater than those observed with either E2 or VitD3. Additionally, when E2 and VitD3 were packaged with our delivery system, the degree of osteogenic induction was not greater than that of the combination treatment but was much greater than those of each of the other components. Estrogen receptors, ERα&β, expressed on the plasma membrane and nucleus of osteoblasts, play pivotal role in osteogenic differentiation of osteoblasts via Wnt, BMP and Akt signaling pathways [[31], [32]]. VitD3 directly affects osteoblast growth and differentiation by binding with Vitamin D Receptor (VDR) [[33], [34], [35], [36]]. It has been demonstrated that mice lacking VDR exhibit retarded growth and severe bone impairment [37]. Moreover, in mature osteoblast-specific VDR-overexpressing mice, the cortical and trabecular bone volumes are increased [38,39]. Thus, VDR plays a central role in VitD3-induced osteogenesis. The application of E2 and VitD3 to MC3T3-E1 cells facilitates osteogenic differentiation of Bone marrow mesenchymal stem cells (BMSCs) [40]. These results demonstrated that VitD3 improved the osteogenic induction of E2 which consistent with our results. However, the underlying mechanism behind the augmented osteogenic induction observed with E2 and VitD3 treatment is not yet fully understood. In Fig. 7A and B, compared with the E2 alone group, the addition of VitD3 in the E2+VD combination group significantly enhanced the upregulatory effect of E2 on ERα&β expression, whereas E2 had no significant effect on the VitD3-induced increase in VDR expression, indicating that VitD3 facilitated E2-induced estrogenic activity through the upregulation of ERα&β receptors. Intriguingly, other researches also indicated that the osteogenic effect of combined E2 and VitD3 were probably caused by increasing expression of ER and VDR [41]. Thus, the increased expression of ER might contribute to distinctly improved osteogenic ability in MC3T3-E1 cells. The Akt signaling pathway, situated downstream of both ER and VDR, serves as a pivotal regulatory hub for osteogenic differentiation [[42], [43], [44], [45], [46], [47], [48], [49], [50]]. Upon activation by E2 or VitD3, the PI3K/Akt axis modulates the activity of key osteogenic transcription factors, such as Runx2 and Osterix, and promotes the expression of bone formation markers including ALP and Osteocalcin [[51], [52], [53]]. Given this, we hypothesized that the co-administration of E2 and VitD3 might increase E2-induced osteogenic effect by effectively amplifying the activation of the PI3K/Akt pathway. In Fig. 7C, the protein level of P-PI3K, P-AKT, P-mTOR significant increase in E2+VD compared with either E2 or VitD3, indicating that VitD3 facilitated E2 to induce PI3K/AKT/mTOR pathway. Though, p-FOXO3 was not distinctly improved, the level of pan FOXO3 was distinctly improved, indicating that E2 and VitD3 facilitate the transcription of FOXO3. To verify the involvement of Akt signaling, Akti1/2 was applied in MC3T3-E1 cells, which significantly inhibited ALP activity in the E2, VitD3, and E2+VD groups (Fig. 7D). The fact that Akt inhibition neutralized the enhanced osteogenic capacity of the E2+VD combination further proves that these two agents improved osteogenesis through the Akt pathway. In summary, when combined with E2, VitD3 can not only upregulate VDR expression but also enhance E2-induced ER expression, thereby activating the downstream PI3K/AKT/mTOR signaling pathway more potently than either agent alone.

**Fig. 7:**
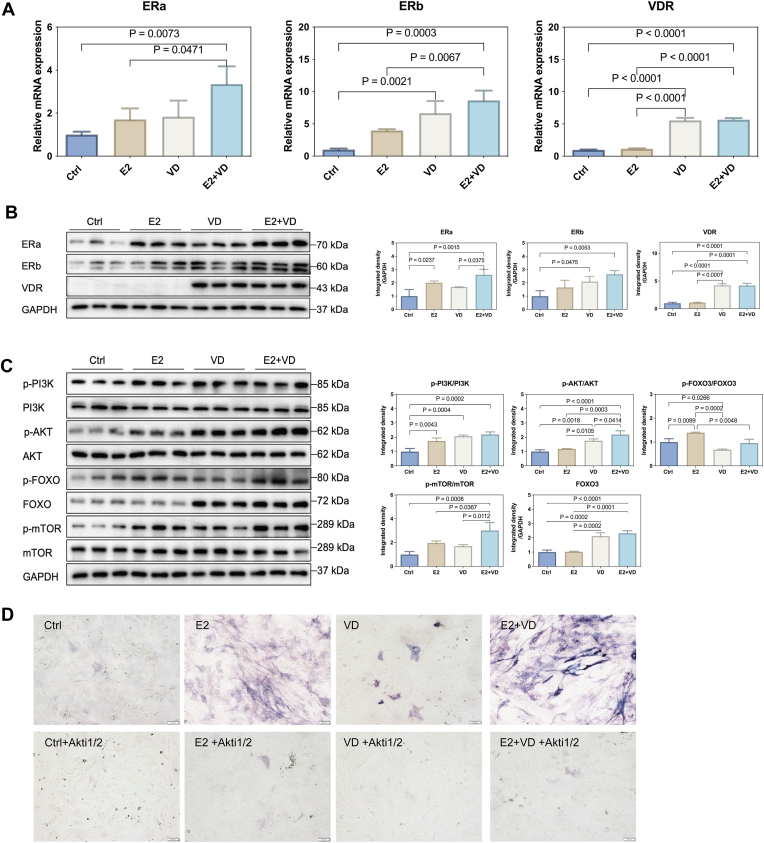
E2 and VitD3 improves osteogenesis by PI3K/AKT/mTOR pathway. (A) Q-PCR analysis of mRNA level of ERα, ERβ and VDR in Ctrl, E2, VD and E2+VD in MC3T3-E1 cells. (B) The protein level of ERα, ERβ and VDR in Ctrl, E2, VD and E2+VD in MC3T3-E1 cells. (C) The WB analysis of protein level of pan and phsopho-PI3K, pan and phospho-Akt, pan and phospho-mTOR, and pan and phospho-FOXO3 in MC3T3-E1 cells treated with PBS, E2, VitD3, E2 plus VitD3. (D) The MC3T3-E1 cells were pretreated with or without Akti1/2 (5uM), and were induced to osteoblasts with PBS, E2, VitD3, E2+VitD3. The ALP staining was utilized to evaluate the osteogenic differentiation in each group.

### 3.5 Safety evaluation of E2+VD@PLGA_IR780_ADA

Biosafety is a critical consideration for clinical application in patients; thus, we tested whether the nanoparticles have underlying toxicity in cells and cause side effects in mice. As shown in Figure S4, E2, the nanocapsule, and E2+VD@PLGA_IR780_ADA did not reduce the viability of MC3T3-E1 cells. E2 is an FDA-approved therapy for the prevention of postmenopausal osteoporosis with added notice: “When prescribing solely for the prevention of postmenopausal osteoporosis, therapy should only be considered for women at significant risk of osteoporosis and for whom nonestrogen medications are not considered to be appropriate.” Current recommendations are to use estrogen for the relief of menopausal symptoms at the lowest dose necessary and for the shortest time possible. All of the above caveats are considered the basis of the side effects caused by E2, such as endometrial hyperplasia, vaginal bleeding, venous thromboembolism (VTE), and cerebrovascular events [54]. ADA was shown to be an effective antiosteoporosis therapy by inhibiting osteoclast-mediated bone resorption. In addition, it was newly indicated as an anchor to bones. Thus, avoiding the side effects of E2 by delivering E2 directly to bones by ADA is an optimal choice. The in vivo distribution of the nanosystem was examined by tracking PBS and E2+VD@PLGA_IR780_ADA by imaging with an in vivo imaging system following intraperitoneal injection into BALB/c mice. Compared with that in the PBS group, significant localization of the nanocarrier to the vertebrae was observed for the E2+VD@PLGA_IR780_ADA group at 8 h postinjection (Fig. 9A). The distribution of E2+VD@PLGA_IR780_ADA within various organs was further evaluated by imaging liver, kidney, spleen, heart, lung, femur, tibia, and fallopian tube tissues at 8 h postinjection. Fluorescence imaging suggested low accumulation of E2+VD@PLGA_IR780_ADA within these organs (figs6, figs7).

**Fig. 8:**
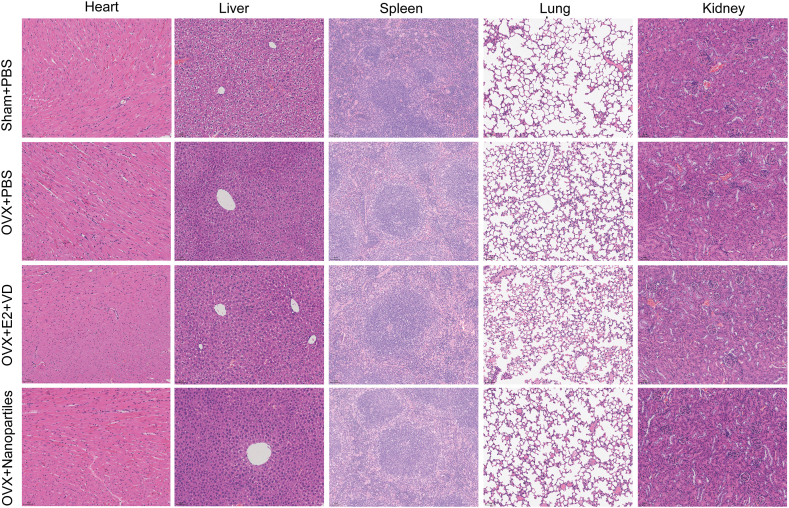
Toxicity of E2+VD@PLGA_IR780_ADA. H&E staining of major organs 24 h after the injection of PBS, E2+VD, or E2+VD@PLGA_IR780_ADA in the Sham and OVX mice.

**Fig. 9:**
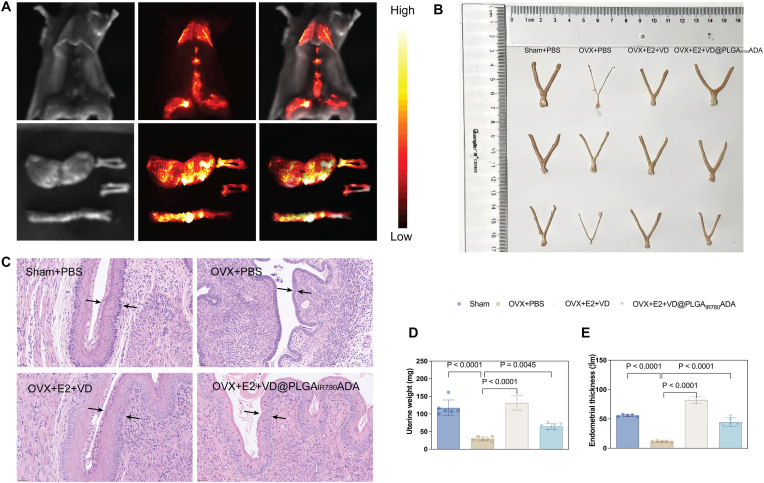
Effects of E2+VD@PLGA_IR780_ADA treatment on the uteri of OVX mice. In situ and ex vivo UCL images of bones. (A) UCL images of E2+VD@PLGA_IR780_ADA 6 h after injection. (B) Whole images of uteri from the Sham + PBS, OVX + PBS, OVX + VD + E2, and OVX + E2+VD@PLGA_IR780_ADA groups. (C) H&E images of uteri from the four groups. (D) Uterine weight was analyzed immediately after uterus separation. (n = 5) (E) Analysis of endometrial thickness by H&E staining of uteri from the 4 groups. (n = 5).

Next, we examined the pathology of the main organs from the Sham + PBS, OVX + PBS, OVX + E2+VD, and E2+VD@PLGA_IR780_ADA groups and evaluated their histology by H&E staining. As shown in Fig. 8, the heart, liver, spleen, lungs, and kidneys did not exhibit any histological anomalies. Importantly, because E2 targets and stimulates the endometrium, we observed the size and weight of the uterus (Fig. 9B–E). As shown in Fig. 9B and D, the size and weight of the uterus were lower in the OVX + PBS group than in the Sham + PBS group, which is in accordance with the shrinking of the uterus observed postmenopause. In the OVX + E2+VD and OVX + E2+VD@PLGA_IR780_ADA groups, the size and weight of the uterus were much greater than those in the OVX + PBS group. Surprisingly, although the uterus was larger in the OVX + E2+VD@PLGA_IR780_ADA group than in the OVX + PBS group, it was much smaller than that in the OVX + E2+VD group. Additionally, H&E staining revealed that the endometrium was thinnest in the OVX + PBS group (Fig. 9C). After E2+VD or E2+VD@PLGA_IR780_ADA was applied to OVX mice, the endometrium grew thicker than that in the OVX + PBS group (Fig. 9E). Consistent with the uterus size and weight, the thickness of the endometrium was much lower in the OVX + E2+VD@PLGA_IR780_ADA group than in the OVX + E2+VD group. The above results demonstrated that E2+VD@PLGA_IR780_ADA is not toxic to major organs and is less stimulating to the endometrium.

## 4 Conclusion

In this study, we synthesized ADA-linked PLGA nanoparticles, E2+VD@PLGA_IR780_ADA, to package E2 and VitD3. E2+VD@PLGA_IR780_ADA completely released E2 and VitD3 within 48 h, and ADA modification facilitated the targeting of osteoblasts to bones in vitro and in vivo. When E2+VD@PLGA_IR780_ADA was applied to MC3T3-E1 cells for differentiation, the osteogenic tests revealed greater ALP activity, osteogenic marker expression, and calcium deposition than did the controls. Compared with OVX, treating OVX mice with E2+VD@PLGA_IR780_ADA significantly improved bone mass, stimulated bone formation, increased BMD, and improved bone microstructure and serum bone turnover markers. Furthermore, E2+VD@PLGA_IR780_ADA prevented E2-induced endometrial stimulation and potential organ toxicity. Mechanistically, the combined application of VitD3 and E2 allows VitD3 to perform two key functions: upregulating VDR expression, and enhancing the E2-induced increase in ER levels, which in turn results in a stronger activation of the PI3K/AKT/mTOR signaling pathway than the use of either molecule individually. In summary, E2+VD@PLGA_IR780_ADA has favorable effects on bone protection both in vitro and in vi vo and might be a novel strategy to improve bone health after osteoporosis.

## CRediT authorship contribution statement

**Yonghui Wang:** Writing – original draft, Software, Project administration, Formal analysis. **Sidi Zhang:** Writing – original draft, Project administration, Investigation, Formal analysis, Conceptualization. **Xinrun Ma:** Writing – original draft, Project administration, Investigation, Formal analysis, Conceptualization. **Donghao Hu:** Methodology, Investigation, Formal analysis. **Junran Liu:** Software, Resources. **Lu Wei:** Methodology, Investigation, Data curation. **Xue Lei:** Methodology, Investigation. **Yan Hu:** Writing – original draft. **Fuyou Li:** Writing – review & editing, Supervision, Resources, Investigation, Conceptualization. **Yanhong Gao:** Writing – review & editing, Validation, Supervision, Funding acquisition, Conceptualization.

## Declaration of competing interest

The authors declare that they have no known competing financial interests or personal relationships that could have appeared to influence the work reported in this paper.

## Figures and Tables

**figs1:**
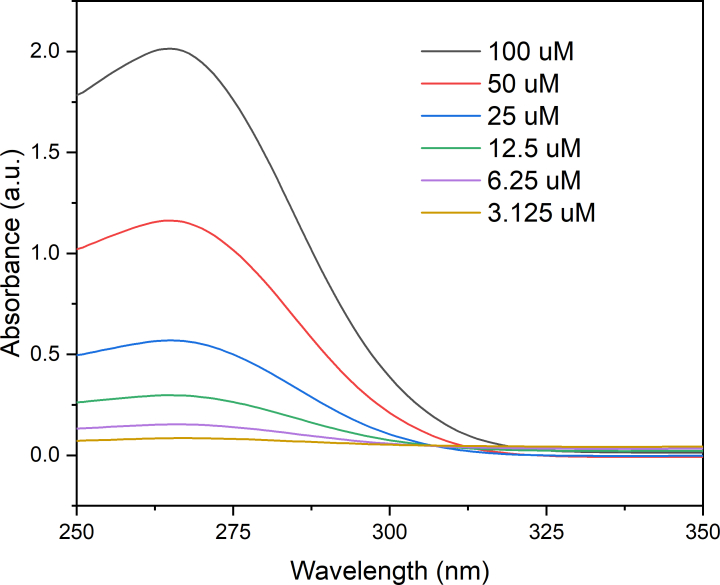
The E2 concentration of E2+VD@PLGA_IR780_ADA was calculated using the absorption spectroscopy. UV-vis absorption spectra of E2 with different concentrations of 31.25-1000 μM.

**figs2:**
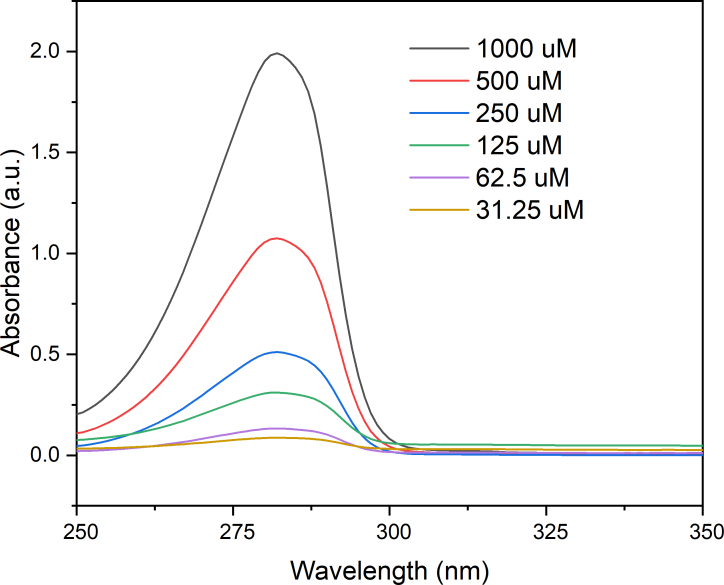
The VD concentration of E2+VD@PLGA_IR780_ADA was calculated using the absorption spectroscopy. UV-vis absorption spectra of VD with different concentrations of 3.125-100 μM.

**figs3:**
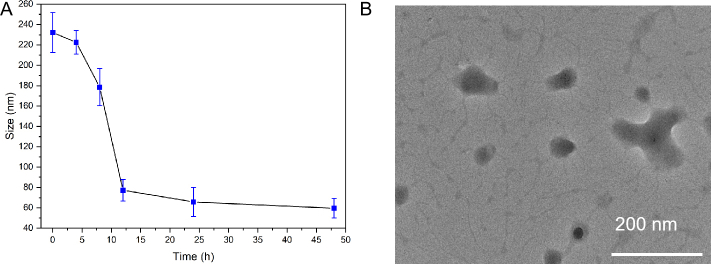
(A) The size changed of E2+VD@PLGA_IR780_ADA during 48 h. (B) the TEM of E2+VD@PLGA_IR780_ADA after 48 h storage in PBS, pH = 7.4.

**figs4:**
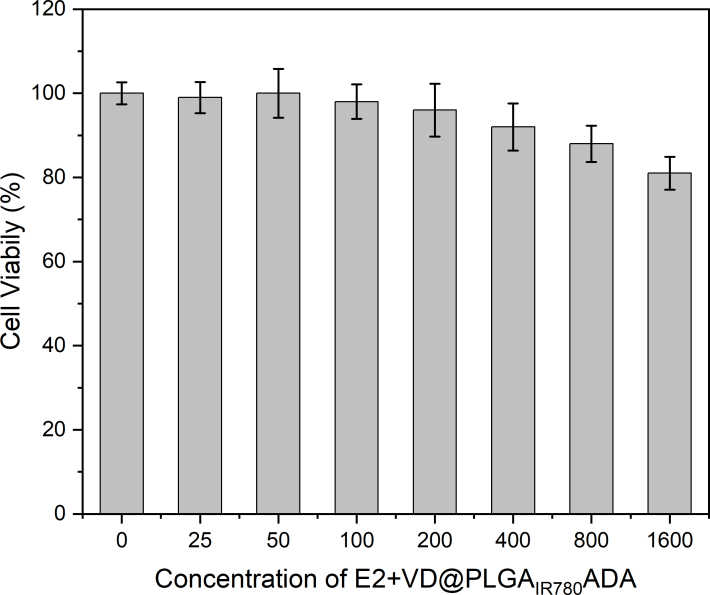
Graph of MTT results of E2+VD@PLGA_IR780_ADA.

**figs5:**
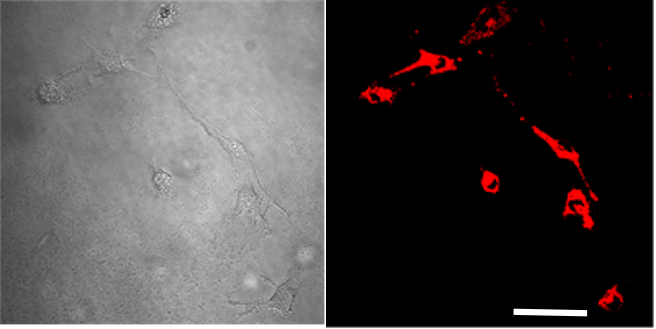
Images of fixed MC3T3-E1 cells incubated with E2+VD@PLGA_IR780_ADA, The scale bar represents 50 μm.

**figs6:**
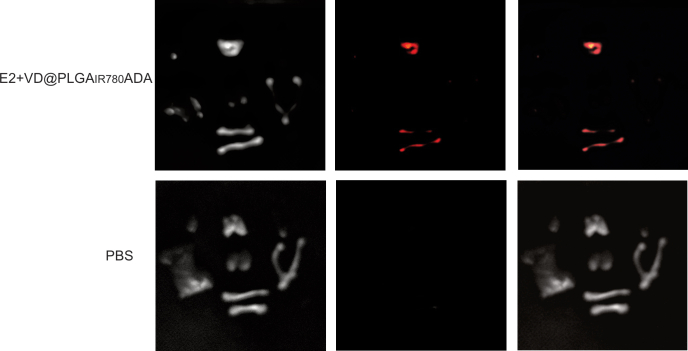
Ex vivo UCL imaging of the mice organs after injection of PBS and E2+VD@PLGA_IR780_ADA.

**figs7:**
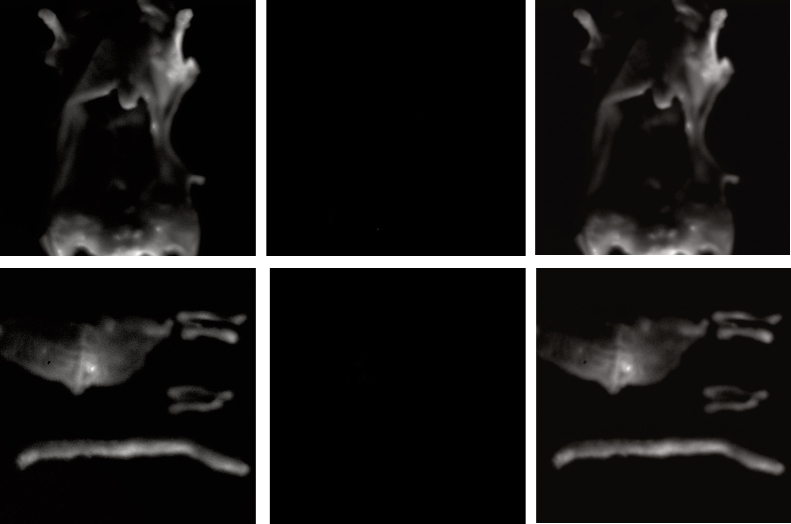
Ex vivo UCL imaging of the mice bones after injection of PBS.

## Data Availability

Data will be made available on request.
