# Supplementary material for: Alendronic acid modified PLGA drug delivery system loaded with 17β-Estradiol and vitamin D3 has anti-osteoporotic effect
Source: Mater Today Bio. 2026 Jan 12;37:102789. doi: 10.1016/j.mtbio.2026.102789 (PMC12854061; doi:10.1016/j.mtbio.2026.102789)
Supplement: Multimedia component 1 [file mmc1.docx]

Table 1. Primers for RT-PCR

| Primer | Sequence (5’-3’) |
| --- | --- |
| ERα-R | \| CAAAGGTTGGCAGCTCTCAT \| \| --- \| |
| ERβ-F | \| GAAGCATTCAAGGACATAATG \| \| --- \| |
| ERβ-R | \| TCCCACTTCGTAACACTTC \| \| --- \| |
| VDR-F | \| GCAACAGCACATTATCGCCATC \| \| --- \| |
| VDR-R | \| ACCAGCTTAGCATCCTGTACCC \| \| --- \| |
| ALP-F | GACTGGTACTCGGATAACGA |
| ALP-R | TGCGGTTCCAGACATAGTGG |
| Runx2-F | TGAGGGATGAAATGCTTGGGAACTG |
| Runx2-R | GATGATGACACTGCCACCTCTGAC |
| IBSP-F | CCGGCCACGCTACTTTCTT |
| IBSP-R | TGGACTGGAAACCGTTTCAGA |
| GAPDH-F | \| AGGTCGGTGTGAACGGATTTG \| \| --- \| |
| GAPDH-R | TGTAGACCATGTAGTTGAGGTCA |
